# Adjustment Processes After the First Wave of the COVID-19 Pandemic: A Grounded Theory Study Based on Clinical Psychologists’ Experience

**DOI:** 10.3389/fpsyg.2022.854745

**Published:** 2022-03-04

**Authors:** Giulia Lamiani, Lidia Borghi, Federica Bonazza, Daniela Rebecchi, David Lazzari, Elena Vegni

**Affiliations:** ^1^Department of Health Sciences, University of Milan, Milan, Italy; ^2^Studi Cognitivi, Modena, Italy; ^3^Unit of Clinical Psychology, Hospital of Terni, Terni, Italy; ^4^Unit of Clinical Psychology, ASST Santi Paolo e Carlo Hospital, Milan, Italy

**Keywords:** clinical psychology and health, adjustment (psychology), COVID-19, qualitative research and analysis, population

## Abstract

**Background:**

Anxiety, depression, and post-traumatic stress have been reported among the general population during the first wave of the COVID-19 pandemic. However, the adjustment after the emergency phase remains under-investigated. This study aims to understand the adjustment processes of the population after the emergency phase of the pandemic.

**Methods:**

We conducted a grounded theory based on the experience of 24 clinical psychologists who provided extensive support to the population during the pandemic in different Italian regions. Three online focus groups were conducted. The transcripts of the focus groups were analyzed through a process of open, axial, and selective coding. Data collection terminated once thematic saturation was reached.

**Results:**

Repositioning emerged as the evolutionary task people were confronted with in the face of a New Reality. Repositioning meant dealing with and integrating unpleasant Emotional Experiences deriving from the lockdown and reopening (i.e., unsafety, emotional exhaustion, loneliness, uncertainty, loss, and disconnection) through different Coping Strategies. Repositioning was facilitated or hindered by contextual and individual Intervening Conditions and led to two Adjustment Outcomes: growth or block.

**Conclusion:**

Results suggest that repositioning was the core task people had to face after the emergency phase of COVID-19. Proactive psychological interventions may support the population in repositioning in order to prevent maladjustment and encourage post-traumatic growth.

## Introduction

The COVID-19 pandemic was declared by WHO a public health emergency of international interest on January 30th, 2020. Since then, it spread rapidly nationwide, affecting over 312,173,462 and 222 countries (World Health Organization, 2022). Italy was the first Western country to be severely affected, with 7,971,068 confirmed cases and 139,872 deaths (Italian Ministry of Health, 2022). At the outbreak of the COVID-19 pandemic, several countries implemented confinement measures such as nationwide lockdowns and quarantines to contain the virus. Because of these measures, people were confronted with several stressors, such as physical isolation, forced cohabitation, impossibility to hold funeral rituals, suspension of schools and social activities, economic losses, and excessive workloads (Pfefferbaum and North, 2020). A large body of research assessed the impact of the COVID-19 pandemic and lockdown measures on the mental health of the population. Quantitative studies and reviews reported a prevalence of anxiety, depression, and post-traumatic stress among the general population, with variations depending on the psychological and contextual resources (Castelli et al., 2020; Morales-Vives et al., 2020; Wang et al., 2020; Prati and Mancini, 2021). For example, Rossi et al. (2020), who explored the psychological stress caused by the pandemic and the lockdown among the Italian population, observed the presence of post-traumatic stress symptoms and adjustment disorders in one-third and one-quarter of the sample, respectively. Similarly, Lenzo et al. (2020) showed that about a third of Italian respondents reported moderate to extremely severe depression, anxiety, and stress.

As many other countries, during the summer of 2020, Italy entered a reopening phase during which business, services, and activities gradually resumed. Reopening was a challenge not only from an epidemiological point of view but also from a psychological standpoint. According to previous studies (Young et al., 2002), in the recovery phase of an emergency, the prevalence of psychological and mental disorders may increase. Despite individual resources and resilience, people may find it difficult to adapt to the new circumstances and integrate the traumatic events into a new narrative with meaning (Kazlauskas and Quero, 2020). Studies from previous epidemics such as HIV, SARS, and Ebola have shown that fear, panic, and stigma might endure among the population even when the disease is normalized (Strong, 1990; Hong et al., 2009; Ji et al., 2017). Despite this evidence, little attention has been paid so far to the adjustment processes of the population after the first wave of the COVID-19 pandemic.

Adjustment has been described as the process through which human beings modify attitudes and behaviors in response to environmental demands or unexpected conditions (American Psychological Association, 2020). In other words, adjustment may be seen as the attempts to maintain a balance between own needs and the circumstances that may impede their satisfaction. The pandemic onset and the subsequent lockdowns have dramatically altered the individuals’ environment worldwide: they radically changed everyday life and challenged the satisfaction of basic human needs, such as physiological, safety, belongingness, and self-actualization needs (Maslow, 1954). A US study (Suh et al., 2021) that analyzed web searched interactions for 14 months starting from January 6, 2020 revealed an increased expression of physiological needs during the pandemic onset compared to the pre-pandemic period. Shifts in the expression of needs were also observed in the period after the lockdowns (Suh et al., 2021). Therefore, it is possible that adjustment processes may have been triggered during the lockdown as well as after the emergency period.

This study aims to understand the adjustment processes of the population after the first wave of the COVID-19 pandemic in Italy. For this purpose, we conducted a grounded theory study involving clinical psychologists who worked in community and hospital psychological services nationwide during the pandemic. We involved clinical psychologists due to their privileged perspective on the population’s psychological distress and their expertise in assessing psychological adjustment processes.

## Materials and Methods

### Research Methodology

We used the grounded theory method (Glaser and Strauss, 1967) to understand the adjustment process after the first wave of the COVID-19 pandemic based on clinical psychologists’ experience. Grounded theory is a qualitative method based on an inductive process through which a theory is derived from the data (Strauss and Corbin, 1990). For this reason, grounded theory is a particularly useful method for understanding unexplored social processes or phenomena, where there is no theory or model to explain them. As the adjustment processes of the population after the first wave of the COVID-19 pandemic remain unknown, grounded theory was chosen as a particularly suitable method for studying this process.

### Participants

The participants were clinical psychologists with training in psychotherapy. Clinical psychologists were recruited nationwide through the National Boards of Psychologists and the regional Departments of Mental Health. The recruiting followed the principles of theoretical sampling (Draucker et al., 2007). Theoretical sampling, as opposed to probability sampling, aims to include information rich cases for in-depth study. As we aimed to explore the adaptation process of the general population after the first wave of COVID-19 pandemic, we selected psychologists with extensive experience in emergency psychology and in providing psychological support during the pandemic. In order to capture the variability of the adaptation processes, we selected psychologists to account for a broad variety of characteristics such as the population they work with (e.g., children and adolescents, families, adults, and chronic patients), the region of Italy they work in, their responsibility role at work, and their psychotherapeutic approach.

### Data Collection

The sampling and data collection were carried out simultaneously. Given the necessary safety measures, the data collection was conducted online. Psychologists interested in the research were sent a link to Surveymonkey platform where they could express their consent and complete a socio-demographic questionnaire. After completion of the questionnaire, participants were e-mailed an invitation to join a focus group *via* Microsoft Teams. The focus group lasted an hour and a half and was open to 8–10 participants.

Three focus groups involving different participants were held in July 2020 by the first (GL) and second author (LB) and were audio-recorded. During the focus groups, GL presented the research, facilitated the participants’ introduction, and led the group discussion. LB co-facilitated and wrote memos of the most salient aspects emerging from the discussion and from personal reflections. During the focus groups, the participants were asked to share their experiences and opinions on two questions: “What are the main psychological challenges that you are now observing in the population you work with?” and “What do you think are the protecting or risk factors of these challenges?” As the data collection and data analysis were conducted simultaneously, three focus groups were held, after which data saturation was achieved.

### Data Analysis

The audio-recordings were transcribed verbatim. All details relating to patients or places were removed. Two researchers (GL, LB) analyzed the anonymized transcripts according to grounded theory principles (Strauss and Corbin, 1990). The analysis was conducted in three stages: open, axial, and analytic coding. In the open coding stage, the researchers independently examined the focus group transcripts for salient categories, applying descriptive codes to the text. The aim of this stage was to fragment the data and delineate an initial list of codes with maximum flexibility and with no theoretical assumptions. The language of the participants guided the development of the codes’ labels. During axial coding, the codes were progressively aggregated into broader categories. In this stage, the researchers met several times to organize the categories by making connections among them and clarifying their relationships (Strauss and Corbin, 1990). To help with this task, Corbin and Strauss (1990) developed a coding paradigm composed of six categories, which are: the phenomenon under investigation, its causal condition, the intervening conditions, the contextual factors that moderate its occurrence, the strategies to deal with it, and the consequences. These categories help ensure that the researchers have fully explored the process under investigation. The relationships between the categories were verified through an iterative process of going back and forth from the data to the coding and vice versa. Once the axial coding was completed, the researchers engaged in selective coding. In this stage of analysis, researchers usually generate a theory from the data. This abstract level of coding requires the identification of a core category that is the pivotal concept that articulates the whole process under investigation. In this phase, a graphical model was created to illustrate the relationship between the core category and the other categories. At the end of each coding stage, the researchers (GL and LB) met with the research team (FB, DR, and EV) through periodical online meetings to discuss the coding and receive feedback on the reliability of the findings.

### Ethics

The study was conducted according to the guidelines of the Declaration of Helsinki and was approved by the Ethics Committee of the University of Milan (study reference number 74.20, approved on June 29th 2020). Informed consent was obtained electronically from all participants involved in the study.

## Results

### Participants

A total of 24 psychologists participated in three focus groups. Their socio-demographic and professional characteristics are reported in Table 1. The participants were mainly females (87.5%), with a mean age of 47 years and with an average of 21 years of clinical experience. Most participants (71%) worked in regions of northern Italy.

**Table 1:** Characteristics of the study participants.

Characteristics	*n* (%)
*Gender*	
Female	21(87.5%)
Male	3(12.5%)
*Age*	
Mean (SD)	47.08(8.77)
Range	32–63
*Years of experience*	
Mean (SD)	20.96(9.5)
Range	4–41
*Psychotherapy orientation*	
Dynamic	8(33.3%)
Systemic	5(20.8%)
Cognitive Behavioral	5(20.8%)
Humanistic	2(8.3%)
Other (i.e., Gestalt psychotherapy)	4(16.8%)
*Italian region*	
Lombardia	6(25%)
Piemonte	3(12.5%)
Veneto	2(8.3%)
Trentino-Alto Adige	2(8.3%)
Emilia Romagna	4(16.7%)
Umbria	3(12.5%)
Sardinia	4(16.7%)
*Working Context*	
Public	20(83.3%)
Association	4(16.7%)
*Working Location*	
Hospital	15(62.5%)
Territory	9(37.5%)
*Population target (more than one option possible)*	
Children/Adolescents	12(50%)
Adults	20(83.3%)
Families	10(41.7%)
Chronic patients	10(41.7%)
*Responsibility role*	
Director	5(20.8%)
Employee	19(79.2%)
*Number of COVID-19 patients supported*	
0–10	1(4.2%)
10–30	6(25%)
> 30	17(70.8%)
*Psychological intervention provided to the population (more than one option possible)*	
Face-to-face psychological support	13(54.1%)
Online psychological support	19(79.1%)
Face-to-face psychotherapy	2(8.3%)
Online psychotherapy	2(8.3%)

### Grounded Theory

**Figure 1:**
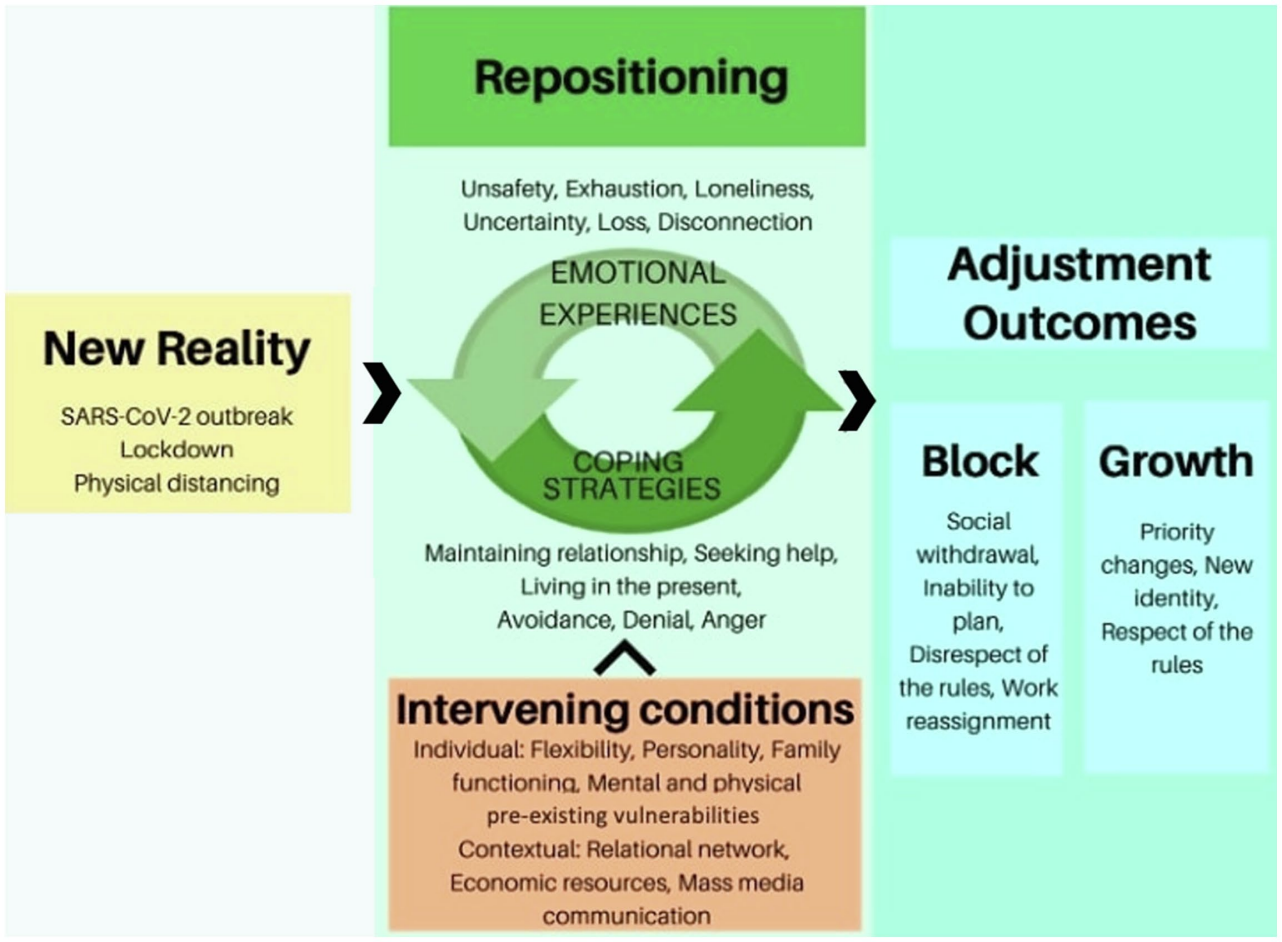
The adjustment process after the first wave of the COVID-19 pandemic.

The adjustment process after the first wave of the COVID-19 pandemic is presented in Figure 1. The analysis revealed that, in the face of a New Reality, repositioning was the core evolutionary task that people had to face in order to resolve the adjustment process. Repositioning required an inner process of integration of the Emotional Experiences caused by the New Reality through Coping Strategies. Repositioning was facilitated or hindered by contextual and individual Intervening Conditions and led to two different Adjustment Outcomes: growth or block. The categories of the model are described below, along with some quotes, by way of example, taken from the transcripts of the focus groups. Quotes are followed by the focus group number in which emerged and by the identification number of the participant [e.g., FG1, participant (part) 1].

#### The New Reality

The COVID-19 outbreak and the consequent safety measures introduced to limit the contagion shaped a new reality and new habits. This new reality has been described “*as though it were a big stone weighing on everyone, even on those who were not directly involved*” (FG1, part 3). According to the participants’ experience, the prolonged lockdown and isolation, physical distancing, reopening, and uncertainty toward the future were all aspects of the new reality that caused suffering among the population and triggered the need for repositioning in the face of the new normal: “F*rom an organisational perspective, it is a very complex period now, because the answer is no longer just ‘no, it’s not possible, the service is closed’, but it is ‘yes, but on the condition that you respect a whole series of protection rules’ and therefore the stress is increased*” (FG 2, part 3).

“*There are cancer patients, especially older ones, who complain because their children, who are worried about them, no longer bring their grandchildren and this is a source of great suffering*” (FG2, part 2).

#### Repositioning

The disruption of the old reality caused several unpleasant emotional experiences, which people attempted to manage through coping strategies with the aim of repositioning themselves within their lives. “*There is this experience of not finding yourself again, not finding your own centre*” (FG 2, part 2). Repositioning emerged as the challenge of giving meaning to the emotional experiences in order to “*readjust after the lockdown, and project into the future, which is not the same as it was before and is still uncertain*” (FG2, part 7).

##### Emotional Experiences

The emotional experiences described were mainly unpleasant. Lack of safety, fear, and anxiety were frequently reported by the population and chronic patients who “*are returning to hospital slowly and reluctantly, still seeing the hospital as dirty, with some degree of danger*” (FG1, part 6). “*Many report fear of sociality, fear in regulating distances, fear to resume a new normal and leave that safe place that is the house to go out to make some errands or to go to work*” (FG3, part 5). Fear of this invisible virus and of the possibility of infecting grandparents was observed among children. Dread that the emergency was not over was also observed among healthcare professionals.

Another emotional experience that emerged was the feeling of physical and emotional exhaustion. Exhaustion was observed among healthcare workers and among caregivers of children, people with disabilities, or chronic patients, who did not receive any support during the lockdown: “*Family members of people with cognitive impairments had to manage situations of increased caregiving burden with a subsequent exhaustion of physical and psychological resources*” (FG1, part 2).

The lockdown experience and physical distancing fostered an experience of loneliness in the population that persisted after the lockdown. Many healthcare professionals lived far from their families for months. Caregivers “*had to manage complex situations without the help of a relative going to do the errand, without being able to have a break, without the closeness of the professional, the healthcare operator*” (FG 1, part 2).

Problems related to experiences of loss emerged among the population. During the first pandemic wave, many suffered the death of loved ones “*without the possibility of saying goodbye, without having funeral rituals, practical support, and physical contact. We saw families who suffered multiple losses. Sometimes losses happened at distance because the patient was transferred to another hospital. Other times the loss of a family member happened when the patient was unconscious and s(he) learned that the wife or the husband had died one month later*” (FG 1, part 1). Some experienced economic, financial, and job losses. The “*feeling that something was lost*” (FG2, part 4) was also present among chronic patients, who felt they were put in a secondary position and among children and adolescents, who felt deprived of the possibility of celebrating the end of a school cycle or the milestone of a graduation.

Another common experience was the feeling of uncertainty, which was not only related to the resurgence of the pandemic but was described as the dramatic experience of existential uncertainty. The pandemic seemed to have deconstructed the sense of omnipotence of medicine but also of life: “*People experienced first-hand that we get sick, and we still die nowadays*” (FG 1, part 6).

Finally, another commonly reported experience was feeling disconnected or extraneous when returning to ordinary life. For many people, the eruption of a new reality, physical isolation, and, in some cases, hospitalization yielded to the sense of disconnection from their relationships and their habits: “*Some patients, especially the oldest ones, are disoriented. Family members are also disconnected because they have not seen each other for three or four months*” (FG2, part 2).

##### Coping Strategies

People used several adaptive and maladaptive strategies to cope with unpleasant emotional experiences. Adaptive strategies included maintaining relationships, seeking help, living in the present, being creative, and integrating past and present experiences. Many people succeeded in reconnecting or maintaining relationships with family members, friends, healthcare professionals, and psychologists despite the physical distancing, thus mitigating the feeling of loneliness. In addition, “*the capacity to reach out for help made the difference. The people who called our (psychological) unit were not feeling well. However, as they were supported, their suffering and its evolution was modulated over time*” (FG3, part 5). The capacity of being in the present and being creative, within the imposed restrictions, was also observed as a positive strategy: “*I noticed that people’s ability to be in the present and to understand that not everything can be controlled was a resource.* S*ome people and families lived more in the present and tried to make sense of this time that has stopped. I am thinking of families who had to stop medically assisted procreation paths or young adolescents who had planned studies abroad*” (FG2, part 3). The possibility of integrating past and present experiences into a narration with meaning emerged as fundamental in order to reconstruct the continuity of self. “*Especially those patients who were in intensive care for a long time, with gaps in their memory, need to rebuild what happened by collecting memories of others and putting the pieces back together*” (FG1, part 7).

Some of the most common maladaptive strategies adopted to protect the self against unpleasant emotional experiences consisted of outward expression of anger, avoidance, denial, controlling behaviors, and alcohol abuse. “*I have seen a lot of anger directed against the institution or on what is external, on the population, on those who did not follow the rules, as if people were looking for a scapegoat on which to offload all this anguish because they did not know what to cling to*” (FG1, part 7). Some people increased controlling behaviors in order to manage anxiety related to the contagion. Others denied the reality of the situation or tried to avoid contact with unpleasant emotional experiences: “*Many clinicians struggle to face death as they have experienced life-threatening situations. Many ask to change jobs”* (FG 3, part 6).

#### Intervening Conditions

Several intervening factors contributed to facilitating or challenging repositioning in the face of a new reality. Some factors were contextual, such as relational networks, economic resources, and mass media communication. The endurance of relationships and social networks, the availability of healthcare services, the timely and proactive support from the psychological services, and the cohesiveness of the teamwork were described as protective factors. For example, for COVID-19, patients having experienced holding and handling relationships with healthcare professionals was crucial: “*There were patients at the field hospital who said ‘those nurses are Russian, they do not speak our language but they massage our feet and we feel taken care of*” (FG1, part 3). The availability of economic and cultural resources was recognized as a protective factor: “*The more the contexts were rich in personal, structural, family, affective and also economic resources, the more resilience there was*” (FG 1, part 6). In addition, mass media communication exposure was described as having an important role in inflating or deflating the perceived severity of the situation: “*When the media started to say that the figures were promising, fear calmed down*” (FG1, part 8).

Other factors were individual, such as personality structure, family functioning, and pre-existing psychological or medical problems. Resilience and psychological flexibility influenced repositioning in the face of the new reality: “*Great resources such as flexibility allowed people not to develop psycho-pathological symptoms. However, there were personality structures that did not hold strong in this phase*” (FG3, part 5). Family functioning and the presence of previous psychological or medical problems modulated the capacity of the population to adapt to the challenges imposed by the pandemic: “*More fragile patients with pre-existing diseases* (e.g.*, cancer or cardiac patients*) *are now experiencing increased suffering*” (FG3, part 2).

#### Adjustment Outcomes

The capacity of repositioning offered people a chance to grow. For some people, this meant changing their priorities and/or assuming a new professional identity: “*For some healthcare professionals the pandemic was a challenge that made them grow, but also redefine their professional identity*” (FG 3, part 2). For other people, adjusting to the new reality meant accepting and respecting the rules to prevent the virus circulation and changing lifestyles.

On the contrary, difficulties in repositioning led people to be stuck in a new present without the capacity of making plans and projecting into a future. Some people reported blocks in their professional activities out of fear or due to unprocessed traumatic experiences: “*I have in mind a patient with excellent motor recovery, who must return to work in a slaughterhouse but has developed the belief that he got the disease directly from the pigs and he cannot ask for a change of role*” (FG 3, part 3). Blocks in social relationships also emerged, with frequent withdrawal. At the same time, oppositional behaviors or non-adherence to the rules emerged, particularly among adolescents: “*Young people continue to stay indoors and to use social media to communicate or play group games*” (FG1, part 2). “*We are also seeing non-adaptive behaviours, such as getting drunk or non-respecting restrictive rules*” (FG 2, part 6).

Finally, failure in repositioning led some people to remain stuck in their previous dysfunctional conditions, such as victims of domestic violence or conflicting families: “*We see women who have great hesitation in following up on complaints. They rethink it or go back, as though the reopening has facilitated the dispersion of that acute conflict that was present in their relationships before and during the lockdown*” (FG 2, part 6).

## Discussion

Although the literature has warned about a possible increase in adjustment disorders as a result of the COVID-19 pandemic (Kazlauskas and Quero, 2020), no research has been conducted to explore the adjustment processes after the emergency phase of the pandemic. This study aimed to understand the adjustment processes after the first wave of COVID-19 pandemic drawing on the experience of clinical psychologists who provided support to the Italian population.

The adjustment process revolved around the core category of repositioning. Repositioning emerged as the evolutionary task that people had to face after being confronted with a disruption of their old reality caused by the COVID-19 outbreak and the lockdown experience. According to adaptation models (Murray Parkes, 1971; Janoff-Bulman, 1989), challenging conditions force people to rebuild their assumptions about the world and the self, consequently transforming the way they interpret the past and expect the future (Janoff-Bulman, 1989). Rebuilding meaning after a traumatic event (de Jong et al., 2020) has been reported as being fundamental to facilitating adjustment. In our study, repositioning was described as an inner work that consisted of integrating and giving meaning to the unpleasant emotional experiences generated by the new reality in order to adjust and move on in life.

Among the unpleasant emotional experiences, anxiety, depressive symptoms, and post-traumatic stress have been widely reported among the general population (Rossi et al., 2020; Favieri et al., 2021) and healthcare professionals (Lamiani et al., 2021; Lasalvia et al., 2021). However, our findings captured some deeper emotional experiences, including feeling unsafe, fear, exhaustion, loneliness, sense of loss, uncertainty, and disconnection. As suggested by other authors (Šakan et al., 2020; Levine et al., 2022), these unpleasant emotions may be a consequence of the frustration of basic psychological needs that occurred during the pandemic. The outbreak of the COVID-19 pandemic and the subsequent lockdown have hindered the satisfaction of the needs of safety, belongingness, and self-actualization threatening the self and its continuity in time, reminding people of their mortality and impotence, and imposing limitations on people’s freedom. Interestingly, these unpleasant emotional experiences did not necessarily develop into psycho-pathological symptoms or maladjustment outcomes thank to individual effective coping strategies or to other intervening factors.

In terms of coping strategies (Lazarus and Folkman, 1984), our findings showed that denial, avoidance, expressing anger, and alcohol abuse were observed by psychologists along with other more functional strategies, such as maintaining relationships, living in the present, being creative, and seeking help. Our findings are consistent with other quantitative studies conducted on the general population, which found that positive thinking, balanced time perspective, active coping style, and social support were positive predictors of psychological well-being during COVID-19 pandemic (Yu et al., 2020; Budimir et al., 2021; Ceccato et al., 2021). Psychological interventions to promote these coping strategies and life skills could be helpful in order to facilitate repositioning and prevent maladjustment outcomes.

Besides individual coping strategies, our findings highlighted that several intervening factors also influenced the capacity of repositioning. Among individual factors, suffering from a chronic or mental health condition before the pandemic and having a rigid personality structure challenged the repositioning work. Our results are consistent with the findings of other recent quantitative studies which found that some personality traits, such as neuroticism and avoidance, and a preoccupied attachment style, are associated with higher psychological distress among the general population and the healthcare professionals (Di Crosta et al., 2020; Mazza et al., 2021). Additionally, one of the most influential factors identified by psychologists in our study was family functioning. An Italian study (Tintori et al., 2020) confirmed that collaboration, affection, and healthy family relationships provided a safe and protective environment during the pandemic. Among contextual factors, we found that mass media exposure, limited economic resources, and the lack of relationships and networks in which people could feel cared for and connected challenged repositioning. On the contrary, the maintenance of family or caring relationships, even *via* the Internet, and the presence of psychological offerings emerged as pivotal factors for preventing maladjustment outcomes.

The success or failure of repositioning in the face of the new reality led to two different adjustment outcomes: growth or block. In line with the literature on post-traumatic growth (Calhoun and Tedeschi, 2001), we know that stressful or traumatic life events may be an opportunity for some people to grow. In our study, the data suggested that as a result of repositioning, some people changed their life priorities, accepted the rules, and resumed their life plans within the limits imposed by the pandemic. On the contrary, others seemed to be blocked in their individual, social, and planning dimensions. Some people did not respect the rules, denying the pandemic situation, others struggled to resume future planning and social relationships, and others still struggled to resume work and asked to be reassigned. Like other stressful events (Murray Parkes, 1971), this pandemic can be conceptualized as a turning point for better or worse psycho-social adjustment. People may have experienced fear, loneliness, uncertainty, loss, and disconnection and may not have been able to make sense of what has happened and to integrate it into their lives. Our findings showed that if such unpleasant emotional experiences are not recognized and integrated, repositioning is challenged and adaptation will probably be inhibited.

This study is qualitative and therefore, its findings have limited generalizability. Moreover, the study is based on the psychologists’ experience in supporting the population and not on the population’s direct experience. We chose to interview psychologists because of their professional knowledge and privileged point of view on the population’s distress and adaptation during the pandemic. However, we are aware that reporting biases may exist. Finally, most participants worked in regions more severely affected by the first wave of the COVID-19 pandemic. This could have created a different psychological impact on the population and on psychotherapists’ experience.

Despite these limitations, our findings may help mental health professionals to proactively plan psychological interventions to prevent maladjustment outcomes. Based on our findings, supportive and therapeutic interventions for the population could facilitate repositioning by encouraging contact with emotional experiences and reinforcing functional coping strategies. Psycho-educational and supportive interventions could be proactively promoted to reach some population targets, such as adolescents, chronic patients, or healthcare professionals, in order to prevent maladjustment (Leone et al., 2020). The results of this study could assist in implementing evidence-based strategies to facilitate the adaptation process during the recovery phase.

## Data Availability Statement

The raw data supporting the conclusions of this article will be made available by the authors, without undue reservation.

## Ethics Statement

The studies involving human participants were reviewed and approved by the Ethics Committee of the University of Milan (study reference number 74.20, approved on June 29th 2020). The participants provided their written informed consent to participate in this study.

## Author Contributions

GL, EV, and DR were involved in the study conceptualization and methodology development. DR involved in the project administration and participants’ recruitment. GL, LB, and FB were involved in data curation and data collection and wrote the original draft of the manuscript. GL, LB, DR, and EV conducted the data analysis. DR, EV, and DL reviewed and edited the manuscript. EV and DL supervised the project. All authors contributed to the article and approved the submitted version.

## Funding

The open access publication was supported by a grant by the University of Milan, Department of Health Sciences (PSR2020_DIP_013_VEGNI).

## Conflict of Interest

The authors declare that the research was conducted in the absence of any commercial or financial relationships that could be construed as a potential conflict of interest.

## Publisher’s Note

All claims expressed in this article are solely those of the authors and do not necessarily represent those of their affiliated organizations, or those of the publisher, the editors and the reviewers. Any product that may be evaluated in this article, or claim that may be made by its manufacturer, is not guaranteed or endorsed by the publisher.
